# The effect of C1-esterase inhibitor on systemic inflammation in trauma patients with a femur fracture - The CAESAR study: study protocol for a randomized controlled trial

**DOI:** 10.1186/1745-6215-12-223

**Published:** 2011-10-11

**Authors:** Marjolein Heeres, Tjaakje Visser, Karlijn JP van Wessem, Anky HL Koenderman, Paul FW Strengers, Leo Koenderman, Luke PH Leenen

**Affiliations:** 1 Department of Trauma Surgery, University Medical Centre Utrecht, Heidelberglaan 100, 3508 GA, Utrecht, The Netherlands; 2 Sanquin Blood Supply Foundation, Plesmanlaan 125, 1066 CX, Amsterdam, The Netherlands; 3 Department of Respiratory Medicine, University Medical Centre Utrecht, Heidelberglaan 100, 3508 GA, Utrecht, The Netherlands

**Keywords:** ARDS, C1-esterase inhibitor, Complication, Femur, Fracture, Inflammation, Interleukin-6, Intramedullary fixation, MODS, Trauma

## Abstract

**Background:**

Systemic inflammation in response to a femur fracture and the additional fixation is associated with inflammatory complications, such as acute respiratory distress syndrome and multiple organ dysfunction syndrome. The injury itself, but also the additional procedure of femoral fixation induces a release of pro-inflammatory cytokines such as interleukin-6. This results in an aggravation of the initial systemic inflammatory response, and can cause an increased risk for the development of inflammatory complications. Recent studies have shown that administration of the serum protein C1-esterase inhibitor can significantly reduce the release of circulating pro-inflammatory cytokines in response to acute systemic inflammation.

**Objective:**

Attenuation of the surgery-induced additional systemic inflammatory response by perioperative treatment with C1-esterase inhibitor of trauma patients with a femur fracture.

**Methods:**

The study is designed as a double-blind randomized placebo-controlled trial. Trauma patients with a femur fracture, Injury Severity Score ≥ 18 and age 18-80 years are included after obtaining informed consent. They are randomized for administration of 200 U/kg C1-esterase inhibitor intravenously or placebo (saline 0.9%) just before the start of the procedure of femoral fixation. The primary endpoint of the study is Δ interleukin-6, measured at t = 0, just before start of the femur fixation surgery and administration of C1-esterase inhibitor, and t = 6, 6 hours after administration of C1-esterase inhibitor and the femur fixation.

**Conclusion:**

This study intents to identify C1-esterase inhibitor as a safe and potent anti-inflammatory agent, that is capable of suppressing systemic inflammation in trauma patients. This might facilitate early total care procedures by lowering the risk of inflammation in response to the surgical intervention. This could result in increased functional outcomes and reduced health care related costs.

**Trial registration:**

clinicaltrials.gov NCT01275976 (January 12th 2011)

## Background

Trauma is a major cause of morbidity and mortality in people under the age of 50 years in the western world [1]. Death can occur as a direct result of the trauma induced injury, or as result of a dysfunctional immune response [2]. This excessive immune reaction is caused by the response to tissue injury, such as seen after trauma, surgery or burns. An overwhelming innate immune response is considered to be a major risk factor in the development of post-traumatic organ failure and sepsis. Additional injury, induced by surgical intervention, can increase the overall immune inflammatory reaction [3].

The lung is most often the first organ to be affected by an exaggerated systemic immune response, which can result in an acute respiratory distress syndrome (ARDS). This functional impairment can be followed by other organs, such as the liver, gastrointestinal tract and kidneys, leading to the so-called multiple organ dysfunction syndrome (MODS). Presence of ARDS and MODS is a major risk factor for mortality, long time morbidity, a prolonged hospital stay and high health care costs [4].

One of the early and systemically released cytokines in the early inflammatory response, is the pro-inflammatory cytokine interleukin-6 (IL-6). Therefore, this cytokine is widely used as an indicator for severity of the systemic inflammatory response in clinical studies [5]. Serum IL-6 levels have been demonstrated to be closely related to the magnitude of the injury (burden of trauma/first hit) and to the operative procedure (second hit) [6,7]. There is a correlation between the IL-6 concentration and the underlying injury severity. Patients with a Injury Severity Score (ISS) > 18 showed a more pronounced rise of IL-6 concentration compared to patients with a lower injury severity [8].

Femur fractures, have been found associated with a profound systemic inflammatory response [9-11]. Ideally, fractures should be managed without a clinically important delay to prevent excess blood loss, and preserve function. However, in case of femur fractures, internal fixation increases systemic inflammation [12]. In trauma patients with an already activated inflammatory response, this increase greatly enhances the risk of an excessive immune response [13,14]. To address this problem, the concept of damage control orthopedics (DCO) was developed [15,16]. This concept aims at minimizing the surgically induced inflammatory response through limiting surgical procedures [16,17]. However, DCO is a controversial approach, because limiting surgical procedures, can lead to a reduced quality of fracture healing, multiple interventions and a prolonged hospital stay. This places the treating surgeon with a difficult dilemma: early total care versus damage control [18-20].

Therefore, there is an unmet need for limiting/preventing the surgical induced inflammation, other than limiting or delaying surgery. Until now, there is a lack of pharmacological interventions that can reduce this surgery induced inflammation.

A promising intervention to attenuate the systemic innate immune response is the treatment with a high concentration of C1-esterase inhibitor (C1-INH) [21]. C1-INH is an acute phase protein, produced by the liver in response to inflammatory conditions. C1-INH is a major inhibitor for both the complement and the contact system, and is, therefore, an important regulator of inflammatory reactions [22,23]. Apart from the modulation of the these systems, C1-INH has also been shown to attenuate systemic inflammation independently of the activation of complement [24]. In fact, Dorresteijn et al showed that administration of C1-INH, in a 'human endotoxemia model', attenuates the release of pro-inflammatory cytokines, including IL-6, in healthy male volunteers [21]. This model evokes a systemic inflammatory response in the absence of complement activation [21,25].

### Aim of the study

The aim of this study is to ascertain whether administration of C1-INH in trauma patients with a femur fracture can reduce the release of pro-inflammatory cytokines and, therefore, will contribute to attenuation of the inflammatory response, in response to a surgical intervention (second hit). This study can provide proof of principle for C1-INH as a potential drug for the prevention of late inflammatory complications in trauma patients.

## Methods

### Objectives

Attenuation of the surgery-induced additional systemic inflammatory response by perioperative treatment with C1-INH in trauma patients with a femur fracture. And the effect of C1-INH on clinical outcome (e.g. ARDS, MODS, mortality, length of hospital stay).

### Study design

This clinical trial is a double blind, placebo-controlled, randomized study, investigating the anti-inflammatory effect of C1-INH on systemic inflammation induced by fixation of the femur fracture in trauma patients.

This study is conducted in accordance with the principles of the Declaration of Helsinki [26] and Good Clinical Practice Guidelines [27]. The independent ethics committee of the University Medical Centre Utrecht (UMCU) approved the study. Written informed consent will be obtained from all participating patients.

### Study population

Seventy multi-trauma patients presented at the emergency department of the UMCU with an ISS ≥ 18 and a femur fracture which need fixation, will be included in the study. Patients are eligible for the study if they meet all the inclusion and none of the exclusion criteria (Table 1).

**Table 1:** Patients in- and exclusion criteria

*Inclusion criteria*	*Exclusion criteria*
Multi-trauma patient ISS* ≥ 18 Femur fracture Age 18-80 years Informed consent	Congenital C1-inhibitor deficiency Use of immune suppressants Known hypersensitivity for blood Products Pregnancy Fixation of the femoral fracture with external fixation or osteosynthesis

*ISS = Injury Severity Score

### Randomization

Subjects are randomly allocated to either the C1-INH group (intervention-group) or the placebo-group just before the start of the surgical repair of the femur fracture, using an 1:1 allocation ratio. Randomization will be performed by the distributing pharmacy (UMCU, The Netherlands) with the use of Design version 2.0 (Systat Software Inc., Chicago, IL, USA). The C1-INH and placebo solutions are prepared in identical non-transparent infusion bags, ensuring the double-blind fashion of the study. Patients, treating surgeons, investigators and nursing personnel involved in the study will be unaware of the randomization, and therefore, the solution applied.

### Study protocol

When trauma patients meet the inclusion criteria, a first blood sample is taken within 12 hours after trauma. This blood sample serves as a reference for the degree of inflammation immediately after injury. Because the first blood sample needs to be drawn within 12 hours after trauma, it is possible that the first sample is drawn without the necessary informed consent and will be analyzed directly. This delayed informed consent may be required because the inflammatory response after the initial trauma is visible in the blood within the first 12 hours after trauma. Of course, the informed consent is obtained as soon as possible if the patient or his/her legal representative is able to. If no consent is obtained, the analyzed blood and the data will be destroyed and the patients will not receive C1-INH or placebo, because the randomization will only take place after informed consent is obtained.

**Figure 1:**
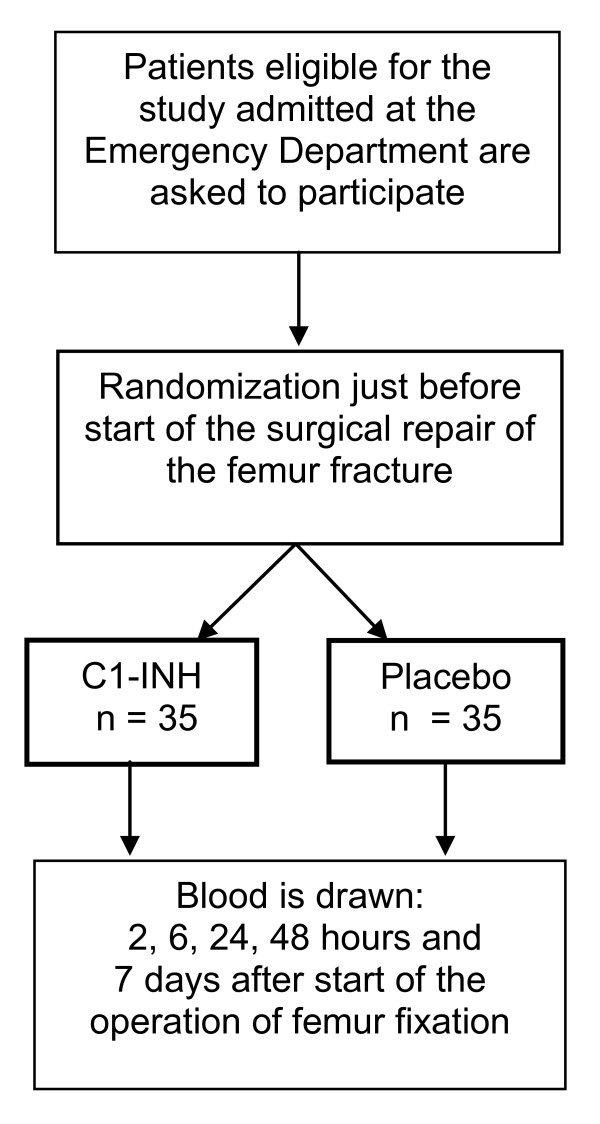
**Time frame**.

Patients receive femur fixation surgery according to the current protocol in the UMCU. Just after induction of anesthesia a second blood sample will be drawn and after this withdrawal the subject receives either C1-INH in a dose of 200 U/kg body weight (n = 35) or placebo (saline 0.9%, n = 35) intravenously. Two hours and six hours after the skin incision for the surgical procedure for femoral fixation the next blood samples are taken. The last samples are taken 24 and 48 hours and seven days after procedure of femoral fixation. An overview of the time frame of the study is shown in Figure 1.

### Outcome measurements

The primary outcome will be the change in serum IL-6 concentration before and after surgical repair of a femur fracture in trauma patients in the presence or absence of C1-INH. We have chosen IL-6 as our primary endpoint because IL-6 is one of the earliest released pro-inflammatory cytokines after trauma, which is detectable by multiplex assays. Several clinical studies have shown that IL-6 is a good marker for the severity of inflammatory response [5,8,10]. Because not all patients are operated at the exact same time after trauma we will use the Δ IL-6 serum concentration (the difference in IL-6 level between t = 0, at the start of the operation, and t = 6, six hours after start of the operation) as our primary endpoint. This allows the detection of the difference between the systemic inflammation in patients treated with or without C1-INH.

Secondary biochemical outcomes include the production of various pro- and anti-inflammatory cytokines, cellular activation markers and complement. Various hematological variables and clinical chemistry measurements, such as haemoglobin, haematocrit, leukocyte count, platelet count, C-reactive protein and fibrinogen, are also determined. These values reflect the clinical condition of the patient and the severity of inflammation.

Secondary clinical outcomes include the effect of C1-INH on the presence or absence of ARDS or MODS and appearance of SIRS, sepsis or septic shock. To evaluate the presence of these clinical conditions the Systemic Response Syndrome (SIRS)-score [28] and the Sequential Organ Failure Assessment (SOFA)-score [29] will be calculated on a daily base during hospitalization of the study subjects. The duration of admission at the intensive care unit, duration of mechanical ventilation and total days of hospital admission are all recorded.

### Sample size calculation

The sample size calculation is based on damping of the increase in serum IL-6 concentration by 30% in the C1-INH group, compared to the placebo group [21,30,31]. With an expected standard deviation of 50% [30,31] and a relevant decrease of IL-6 concentration of 30% [21,30,31], 35 patients in each sample-group (C1-INH or placebo) are needed to find a statistically significant difference (power 80%, Type 1 error rate 0.05).

### Statistical analysis

The primary endpoint, the Δ IL-6, will be determined with the use of a Students' *t-test*. The occurrence of inflammatory complications, such as MODS and ARDS, will be examined with the use of a survival analysis, such as Kaplan Meier or Cox proportional Hazard [32].

For comparisons, a *t-test *or Mann Whitney U-test will be used as appropriate.

Changes over time will be analyzed using repeated measurement analysis with time as within factor and treatment as between factor, using Analysis of Variance.

A *p*-value < 0.05 is considered statistically significant.

The data will be analyzed using software programs SPSS version 17.0 (SPSS Inc., Chicago, IL, USA) and GraphPad Prism (GraphPad Software Inc., La Jolla, CA, USA).

### Interim analysis

In this study there will be two analyses performed, one interim and the final analysis, using the O'Brien Fleming method [33]. The alpha used for the interim analysis will be 0.0054, and for the final analysis the alpha used will be 0.0492.

The interim analysis will be conducted by an independent Data Safety Monitoring Board (DSMB) after the inclusion of 35 succeeding patients, The DSMB is composed of three independent members, of which two clinicians and one statistician. After the interim analysis the DSMB is able to make recommendations. In case of clear benefit, harm or futility of the treatment, the DSMB might decide to end the study early.

## Discussion

This study represents a novel therapeutic approach for the attenuation of the inflammatory response in trauma patients undergoing surgical intervention. To our knowledge this is the first randomized, placebo-controlled trial examining the effect of the acute-phase protein C1-INH on the suppression of the dysfunctional inflammatory reaction in trauma patients during fixation of their femur fracture. Our research, focusing on the potential therapeutic effect of C1-INH, could have important impact on outcome of patients after a severe trauma.

C1-INH is proven to be effective as treatment for improving the outcome in a variety of inflammatory disease models [22,24], including SIRS induced by infusion of LPS [21].

The dosage C1-INH of 200 U/kg bodyweight is distinct from earlier clinical studies. It is shown that C1-INH synthesis increases up to 2.5 times the normal rate during an acute phase response [34,35]. Various states of severe inflammation, such as found during sepsis, burns and ARDS, give a rise in the consumption of C1-INH [36]. Nuijens and colleagues also showed a reduction in functional C1-INH in patients with sepsis complicated by shock or ARDS [37]. Caliezi et al claimed that this decrease in C1-INH levels, as result of the consumption, is probably due to a relative deficiency in functional C1-INH as result of enzymatic cleavage in inflamed or ischemic tissue [22].

We also expect consumption of C1-INH in the severely injured trauma patients in our study and thus a relative deficiency in functional C1-INH. We hypothesize that this consumption is mainly due to (i) the trauma itself, (ii) the blood loss during trauma, (iii) the possible extra blood loss during operation, and (iv) the dilution of the blood compartment during resuscitation because of the use of e.g. saline infusion or packed cells (erythrocytes). To compensate this natural consumption of C1-INH in our patients, we will administer a dose of 200 U/kg bodyweight, approximately a dose of 14.000-16.000 U per patient. This dose is expected to increase the circulating C1-INH concentration at least as found under acute phase conditions [21].

C1-INH is found to be well tolerated up to a dose of 19.000 units in patients suffering from acute myocardial infarction [38]. In the study of Strüber et al a dose of 15.000 U, followed by 7.500 U and 5.000 U (total of 27.500 U) was administered and tolerated without any side effects [39].

It is anticipated that this study will take three years to complete. The study is expected to start in October of 2011 and will end after the last blood sample is drawn from the seventieth successful included patient.

## Conclusion

To the best of our knowledge this study is the first randomized controlled trial designed to assess the use of C1-INH as a possible drug for attenuation of the inflammatory response in trauma patients after a second hit.

And, if our hypothesis is proved correct, it will result in increased functional outcome in trauma patients and reduced health care related costs.

## Trial status

Start trial October 2011, no patients included yet.

## Abbreviations

ARDS: Acute Respiratory Distress Syndrome; C1-INH: C1-esterase inhibitor; DCO: Damage Control Orthopedics; DSMB: Data Safety Monitoring Board; IL-6: Interleukin-6; ISS: Injury Severity Score; MODS: Multiple Organ Dysfunction Syndrome; SIRS: Systemic Inflammatory Response Syndrome; SOFA: Sequential Organ Failure Assessment; UMCU: University Medical Centre Utrecht

## Competing interests

A. Koenderman and P. Strengers both work for Sanquin Blood Supply Foundation. The remaining authors declare to have no competing interests.

## Authors' contributions

MH, TV, LK and LPHL were involved in developing the original study design. MH, LK and LPHL developed the research protocols. AHLK and PFWS developed the design for the study medication. KJPW and LPHL are responsible for the clinical input. MH, LK and LPHL drafted the paper. All authors provided input into revisions of the paper and have approved the final manuscript.
